# Role of MCP-1 and CCR2 in ethanol-induced neuroinflammation and neurodegeneration in the developing brain

**DOI:** 10.1186/s12974-018-1241-2

**Published:** 2018-07-05

**Authors:** Kai Zhang, Haiping Wang, Mei Xu, Jacqueline A. Frank, Jia Luo

**Affiliations:** Department of Pharmacology and Nutritional Sciences, University of Kentucky College of Medicine, 132 Health Sciences Research Building, 1095 Veterans Drive, Lexington, KY 40536 USA

**Keywords:** Alcohol abuse, Apoptosis, Chemokines, Development, Glia, Neurodegeneration

## Abstract

**Background:**

Neuroinflammation and microglial activation have been implicated in both alcohol use disorders (AUD) and fetal alcohol spectrum disorders (FASD). Chemokine monocyte chemoattractant protein 1 (MCP-1) and its receptor C-C chemokine receptor type 2 (CCR2) are critical mediators of neuroinflammation and microglial activation. FASD is the leading cause of mental retardation, and one of the most devastating outcomes of FASD is the loss of neurons in the central nervous system (CNS). The underlying molecular mechanisms, however, remain unclear. We hypothesize that MCP-1/CCR2 signaling mediates ethanol-induced neuroinflammation and microglial activation, which exacerbates neurodegeneration in the developing brain.

**Methods:**

C57BL/6 mice and mice deficient of MCP-1 (MCP-1^−/−^) and CCR2 (CCR2^−/−^) were exposed to ethanol on postnatal day 4 (PD4). Neuroinflammation, and microglial activation, and neurodegeneration in the brain were evaluated by immunohistochemistry and immunoblotting. A neuronal and microglial co-culture system was used to evaluate the role of microglia and MCP-1/CCR2 signaling in ethanol-induced neurodegeneration. Specific inhibitors were employed to delineate the involved signaling pathways.

**Results:**

Ethanol-induced microglial activation, neuroinflammation, and a drastic increase in the mRNA and protein levels of MCP-1. Treatment of Bindarit (MCP-1 synthesis inhibitor) and RS504393 (CCR2 antagonist) significantly reduced ethanol-induced microglia activation/neuroinflammation, and neuroapoptosis in the developing brain. MCP-1^−/−^ and CCR2^−/−^ mice were more resistant to ethanol-induced neuroapoptosis. Moreover, ethanol plus MCP-1 caused more neuronal death in a neuron/microglia co-culture system than neuronal culture alone, and Bindarit and RS504393 attenuated ethanol-induced neuronal death in the co-culture system. Ethanol activated TLR4 and GSK3β, two key mediators of microglial activation in the brain and cultured microglial cells (SIM-A9). Blocking MCP-1/CCR2 signaling attenuated ethanol-induced activation of TLR4 and GSK3β.

**Conclusion:**

MCP-1/CCR2 signaling played an important role in ethanol-induced microglial activation/neuroinflammation and neurodegeneration in the developing brain. The effects may be mediated by the interaction among MCP-1/CCR2 signaling, TLR4, and GSK3β.

## Background

Fetal alcohol spectrum disorders (FASD) are a spectrum of defects that can present as physical, mental, and\or behavioral disabilities that stem from ethanol exposure during fetal development. FASD is estimated to affect as many as 2–5% of the population and costs the USA more than $5 billion annually [1]. One of the most devastating effects of developmental exposure to ethanol is the permanent loss of neurons in the central nervous system (CNS) [2, 3]. Although significant progress has been achieved in this line of research, the underlying molecular mechanisms remain unclear. Ethanol-induced neuronal death is accompanied by microglial activation and neuroinflammation [4–7]. Monocyte chemoattractant protein 1 (MCP-1), also called chemokine (CC motif) ligand 2 (CCL2), is a key chemokine involved in neuroinflammation [8–10]. In mouse and human brain, MCP-1 and its receptor CCR2 are primarily expressed by microglia [8]. Although inflammatory response represents one of the first immune processes that protect an organism against diseases following injury, prolonged and sustained inflammation may have cytotoxic effects, aggravating the incidence and the severity of the disease. Increased MCP-1 expression and microglial activation have been observed in the brain of human alcoholics [11]. It has been hypothesized that ethanol exposure may sensitize the immune system to subsequent insults and lead to excess neuroinflammation in alcohol use disorders (AUD) and FASD [12, 13]. It is also proposed that microglia primed by ethanol exposure may contribute to neurotoxicity observed in AUD and FASD [12, 13]. Elevated MCP-1 levels have been observed in brains which undergo various pathogenesis processes, such as multiple sclerosis [14, 15], stroke [16, 17], and Alzheimer’s disease patients [18, 19], and it is believed that the upregulation of MCP-1 is involved in the progression of these diseases [15, 16, 19]. Interestingly, the detrimental effects caused by overexpression of MCP-1 in those diseases were not observed in mice lacking CCR2 [20–23]. Therefore, MCP-1/CCR2 signaling may play an important role in neuroinflammatory diseases. In this study, we sought to determine whether MCP-1/CCR2 signaling is involved in ethanol-induced microglial activation and neuroinflammation in the context of neurodegeneration in the developing brain. We used a well-established third trimester equivalent mouse model in which ethanol exposure caused wide spread neurodegeneration. We showed that inhibiting MCP-1/CCR2 signaling significantly reduced ethanol-induced microglial activation/neuroinflammation and ameliorated neurodegeneration in the developing brain. It appeared that ethanol-induced microglial activation/neuroinflammation was mediated by the interaction among MCP-1/CCR2 signaling, TLR4, and GSK3β.

## Methods

### Reagents

Bindarit, an inhibitor of MCP-1 synthesis, was purchased from Cayman chemical (Ann Arbor, MI, USA). CCR2 antagonist RS504393 was purchased from TOCRIS, Inc. (Minneapolis, MN, USA). TLR4 inhibitor TAK242 was purchased from EMD Millipore, Inc. (Burlington, MA, USA). GSK3β inhibitor SB-216763 was purchased from Sigma-Aldrich, Inc. (St. Louis, MO, USA). Anti-MCP-1 antibody was purchased from Bio-Rad AbD Serotec, Inc. (Raleigh, NC, USA). Anti-CCR-2 antibody was purchased from BioVision, Inc. (Milpitas, CA, USA). Anti-Iba-1 antibody was purchased from Wako Chemicals USA, Inc. (Richmond, VA, USA). All other antibodies were purchased from Cell Signaling Technology, Inc. (Beverly, MA, USA). Other chemicals/reagents used in this project were obtained from Thermo Fisher Scientific, Inc. (Waltham, MA, USA) unless stated otherwise.

### Animals and ethanol exposure

C57BL/6J mice were obtained from Jackson Laboratory (Bar Harbor, ME, USA). C57BL/6 mice, MCP-1^−/−^ (B6.129S4-Ccl2^tm1Rol^/J), and CCR2^−/−^ (B6.129S4-Ccr2^tm1Ifc^/J) mice were obtained from Jackson Laboratories (Bar Harbor, ME, USA). All animals were housed in a specific pathogen-free room within the animal facilities at the University of Kentucky, and handled according to the Institutional Animal Care and Use Committee (protocol #: 2008-0401).

**Fig. 1:**
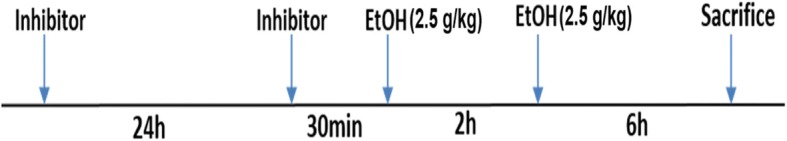
Paradigm for ethanol and inhibitor administration. Mouse pups received two subcutaneous (SC) injections of ethanol which were 2 h apart; each contained ethanol (2.5 g/kg, 20% solution in saline). Bindarit (100 mg/kg) or RS504393 (1 mg/kg) was administered by two SC injections at 24 and 0.5 h prior to the first ethanol exposure

Postnatal day 4 (PD4) mouse pups of either sex from six litters were weighed, and randomly assigned to one of six groups: control, ethanol (EtOH), Bindarit, RS504393, EtOH plus Bindarit, and EtOH plus RS504393. Pups received a total of 5 g/kg ethanol in two subcutaneous (SC) injections which were 2 h apart; each contained ethanol (2.5 g/kg, 20% solution in saline) [24, 25]. We have previously determined that the blood alcohol concentration (BAC) was 338 mg/dl 8 h after the first injection [25]. BACs greater than 300 mg/dl are not uncommon in human alcoholics [26–29]. Some alcoholics with a BAC > 400 mg/dl were coherent and able to drive [29]. BACs> 500 mg/dl have been reported in heavy drinkers [27, 28, 30]. Bindarit is an inhibitor of MCP-1 synthesis and RS504393 is an antagonist for CCR2; they have been extensively used to block MCP-1/CCR2 signaling in vitro and in vivo [31, 32]. Bindarit (100 mg/kg) or RS504393 (1 mg/kg) were administered by two subcutaneous (SC) injections at 24 and 0.5 h prior to ethanol exposure. The concentrations of Bindarit and RS504393 used in this study were based on previous publications [31, 32]. The control pups received an injection of equal volume of saline or DMSO, depending on whether it was an inhibitor or ethanol exposure. At 8 h after the first ethanol injection, pups were sacrificed and the brains were dissected and processed for further analysis. A diagram showing the paradigm of ethanol and inhibitor administration is demonstrated (Fig. 1).

### Culture of microglial cells and neurons

Immortalized mouse microglia cells (SIM-A9) were purchased from American Type Culture Collection (ATCC) (Manassas, VA, USA). SIM-A9 cells are a spontaneously immortalized microglial cell line that exhibits key characteristics of cultured primary microglia [33]. SIM-A9 cells were maintained in Dulbecco’s modified Eagle’s medium (DMEM)/F12 medium supplemented with 5% horse serum, 10% fetal bovine serum (FBS), 2 mM L-glutamine, 100 U/ml penicillin, and 100 μg/ml streptomycin at 37 °C in 5% CO_2_ in a humidified atmosphere. The culture medium was replaced with DMEM/F12 medium without FBS at 50–60% confluence overnight before indicated treatment. The cultures were exposed to ethanol (0.2, 0.4, or 0.8%) in sealed containers. The containers were placed in a humidified environment and maintained at 37 °C with 5% CO_2_. Using this method, the ethanol concentration in the culture medium can be accurately maintained [34]. To block MCP-1/CCR2 signaling, SIM-A9 cells were exposed to Bindarit (300 μM) or RS504393 (100 μM) for 12 h prior to ethanol treatment. The concentrations of Bindarit and RS504393 were based on previous publications [35, 36]. To block TLR4 or GSK3β signaling, SIM-A9 cells were exposed to TAK242 (1 μM) or SB 216763 (10 μM) for 12 h prior to ethanol treatment. The concentrations of TAK242 and SB 216763 were based on previous studies [37, 38]. Primary cortical and cerebellar neurons were generated from the brain of C57BL6 mice on postnatal day 1. The methods for the isolation and culture of primary cortical and cerebellar neurons have been previously described in detail [39, 40]. Briefly, the pups were decapitated, and the brain immediately transferred into dissection medium (97.5% Hank’s balanced salt solution, 0.11 mg/ml sodium pyruvate, 0.1% glucose, 10 mM HEPES, 100 U/ml penicillin, and100 μg/ml streptomycin). The meninges were removed, and the cerebral cortices and cerebella dissected. Cerebral cortical tissues and cerebella were dissociated with 0.25% trypsin followed by 0.1% DNase treatment. Then, tissues were carefully triturated, and the cell suspension was mixed with 4% bovine serum albumin and centrifuged. The cell pellet was resuspended in Neurobasal/B27 medium containing B27 (2%), glutamine (1 mM/L), penicillin (100 U/ml), and streptomycin (100 μg/ml). Cells were plated onto poly-D-lysine (50 μg/ml)-coated cell culture plates. The co-cultures of microglia cells and primary cortical neurons or cerebellar neurons were established in 24-well cell culture plates with inserts as described previously [41]. The cortical or cerebellar neurons were maintained in the cell culture plate containing 500 μl of medium at a density of 4 × 10^6^ cells/well. SIM-A9 cells were grown in a Falcon™ Cell Culture Insert (1 μm pore size, Cat#: C353104) in a separate plate at a density of 1 × 10^6^ cells/per insert in 200 μl of medium overnight at 37 °C and 5% CO_2_, then treated with Bindarit (300 μM) or RS504393 (100 μM) for 12 h. After that, the inserts containing SIM-A9 cells were placed into the wells containing the cortical neurons or cerebellar neurons. The co-cultures were then treated with ethanol for 48 h. The viability of neurons was determined by a 3-(4,5-dimethyl-thiazol-2-yl)-2,5-diphenyltetrazolium bromide (MTT) assay as previously described [40].

### Immunoblotting

After treatment, mice were anesthetized by intraperitoneal injection of ketamine/xylazine, and the cerebral cortices and cerebella were immediately dissected. The tissue was frozen in liquid nitrogen and stored at − 80 °C. The protein from brain tissue or SIM-A9 cells were extracted and processed for immunoblotting (IB) as previously described [40]. Briefly, tissues or cells were homogenized in an ice cold lysis buffer containing 50 mM Tris–HCl (pH 7.5), 150 mM NaCl, 1 mM EGTA, 0.5% NP-40, 0.25% SDS, 1 mM PMSF, 5 μg/ml leupeptin, and 5 μg/ml aprotinin. Homogenates were centrifuged at 20,000 g for 30 min at 4 °C, and the supernatant fraction was collected. Aliquots of the protein samples were separated on a SDS-polyacrylamide gel by electrophoresis. The separated proteins were transferred to nitrocellulose membranes. The membranes were probed with primary antibodies overnight at 4 °C. The immune complexes were detected by the enhanced chemiluminescence substrate (GE Healthcare, Chalfont, Buckinghamshire, UK). The density of immunoblotting was quantified with the software of Image lab 5.2 (Bio-Rad Laboratories, Hercules, CA, USA).

### Quantitative real-time RT-PCR

After ethanol treatment, mice were decapitated and the cortex was quickly frozen in liquid nitrogen and then stored in − 80 °C until use. Total RNA was isolated using TRIZOL reagent (Invitrogen) according to the manufacturer’s instructions. 1 μg of total RNA was used for first strand cDNA synthesis (Promega, A3500). Quantitative real-time RT-PCR was performed on a Lightcycler 480 system (Roche) using a Power SYBR Green PCR Master kit (Invitrogen, 4368706) with cDNA and primers (1 μM) according to the manufacturer’s recommendation. The primers used for analysis were as follows: CCR2 forward 5′-GGTCATGATCCCTATGTGG-3′. CCR2 reverse 5′-CTGGGCACCTGATTTAAAGG-3′. MCP-1 forward 5′-CTTCTGGGCCTGCTGTTCA-3′. MCP-1 Reverse 5′-CAGCCTACTCATTGGGATCA-3′. After finishing the last cycle, a melting curve analysis was performed. Standard −ΔΔCt method was used for determining the gene expression.

### Immunohistochemistry and determination of activated microglia

The procedure for immunohistochemistry (IHC) has been previously described [42]. Briefly, the pups were decapitated and the brain tissues were removed, post fixed in 4% paraformaldehyde for 24 h and then transferred to 30% sucrose in PBS until the brain sunk to the bottom. Sagittal brain sections (20–40 μm) were cut on a freezing microtome. Floating sections were permeabilized with 0.1% Triton X-100 in PBS and incubated in 0.3% H_2_O_2_/50% methanol in PBS and then mounted on slides and dried. The slides were blocked with 5% normal goat serum containing 0.5% Triton X-100 in PBS at room temperature, and then incubated with primary antibodies (diluted in PBS with 1% BSA) overnight at 4 °C. The dilution for the primary antibodies was anti-cleaved caspase-3 antibody, 1:600; anti-Iba1 antibody, 1:600. After washing with PBS, slides were incubated with a biotin-conjugated goat anti-rabbit secondary antibody (1:1000) for 1 h at room temperature and then washed with PBS. Avidin-biotin-peroxidase complex was prepared according to the manufacturer’s instructions. The slides were incubated in the complex for 1 h at room temperature. After rinsing, the slides were developed in 0.05% 3, 3′-diaminobenzidine (DAB) containing 0.003% H_2_O_2_ in PBS.

Iba-1 IHC has been extensively used to identify microglia. Activated microglia were determined morphologically. The resting microglia exhibit long branching processes and a small cellular body, while the activated microglia have fewer but thicker processes with a larger cell body [43]. The average numbers of activated Iba-1-positive cells and all Iba-1-positive cells were calculated from three randomly selected microscopic fields, and three consecutive sections were analyzed for each brain.

### Fluoro-Jade C staining

Fluoro-Jade C is a sensitive fluorescent marker for degenerating neurons. The procedure for Fluoro-Jade C staining has been previously described in detail [44]. Briefly, frozen slides of brain tissue were prepared at a thickness of 10 μm and first immersed in a basic alcohol solution consisting of 1% sodium hydroxide in 80% ethanol. They were then rinsed in 70% ethanol and incubated in 0.06% potassium permanganate solution. Slides were then transferred to a 0.0001% solution of Fluoro-Jade C (Chemicon, Temecula, CA, USA), dissolved in 0.1% acetic acid vehicle. The slides were then rinsed with distilled water. The air-dried slides were then cleared in xylene for at least 1 min and then cover slipped with DPX (Sigma) non-fluorescent mounting media. The slides were examined and recorded with a fluorescent microscope (IX81, Olympus).

## Statistical analysis

The data were presented as mean ± SEM. Statistical significance was determined by the ANOVA followed by Tukey’s post hoc test. Differences in which *p* was < 0.05 were considered statistically significant.

## Results

### Ethanol increases MCP-1 expression in the developing brain and cultured microglial cells

**Fig. 2:**
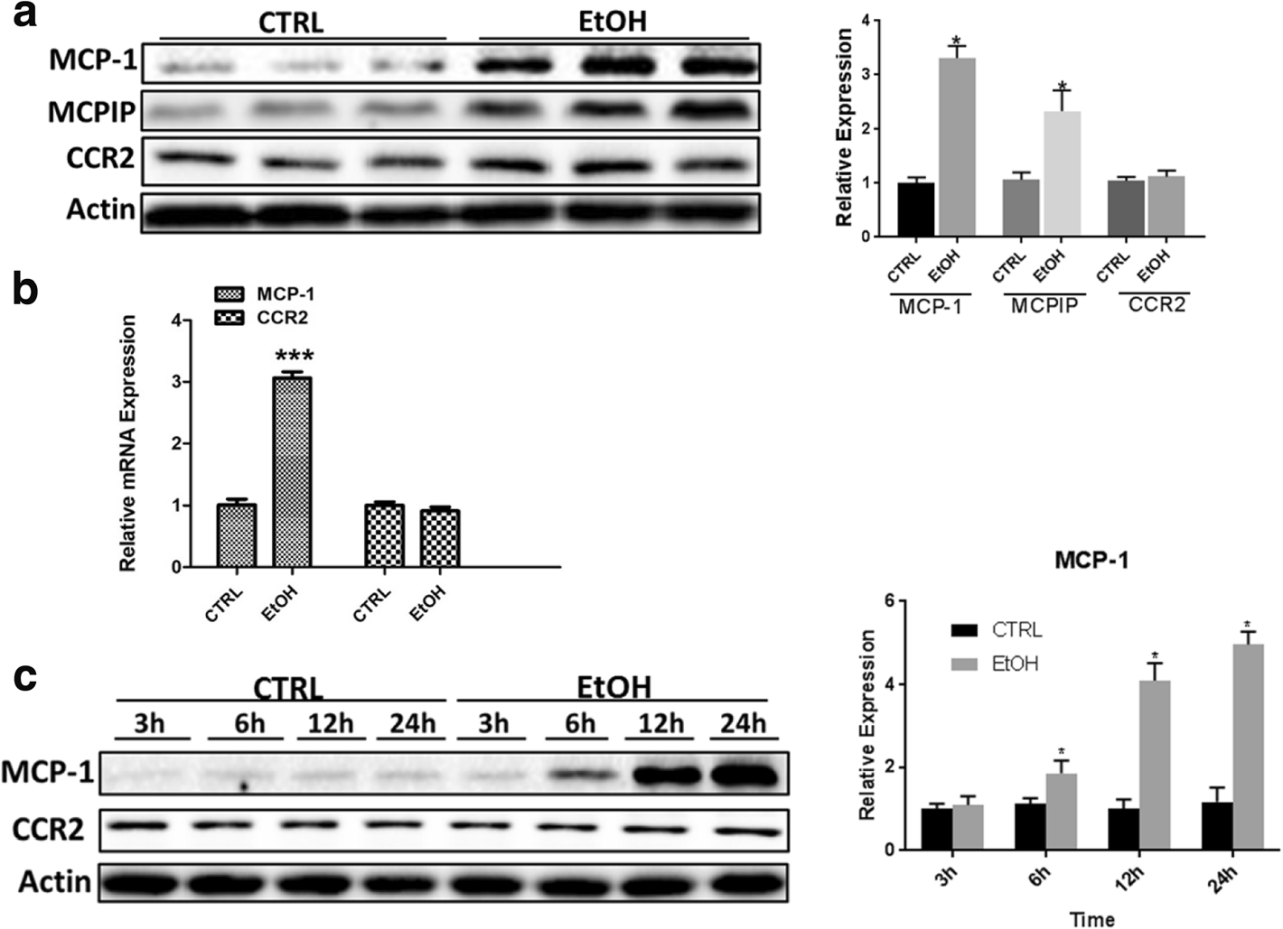
Effect of ethanol on MCP-1 and CCR2 expression. **a** C57BL6 mice of postnatal day 4 (PD4) were exposed to ethanol (EtOH) as described in the “Methods” section. 8 h after EtOH exposure, mice were sacrificed and the brain was processed for immunoblotting (IB) analysis of MCP-1 and CCR2. The expression of MCP-1, MCPIP, and CCR2 in the brain was quantified and normalized to actin. Each data point was the mean ± SEM of six animals. **b** The expression of MCP-1 and CCR2 mRNA in the brain was determined by quantitative RT-PCR as described in the “Methods” section. Each data point was the mean ± SEM of three animals. ***p < 0.001, statistically significant difference from control group. **c** SIM-A9 cells were exposed to EtOH (0.4%) for 3, 6, 12, or 12 h. The expression of MCP-1 and CCR2 was determined by IB and quantified by the normalization to the expression of actin. Each data point was the mean ± SEM of three independent experiments. *p < 0.05, statistically significant difference from control group

Ethanol exposure increases the expression MCP-1 in the adult brain of mice and human alcoholics [11, 45]. To evaluate the role of MCP-1/CCR2 in ethanol-induced damage in the developing brain, we first sought to determine the effect of ethanol on MCP-1/CCR2 expression in the developing brain. Using a well-established mouse model of postnatal ethanol exposure [2, 24, 25], we showed that ethanol increased MCP-1 expression but not CCR2 in the brain of postnatal day 4 (PD4) mice (Fig. 2a). Ethanol also upregulated MCPIP, a down-stream effector of MCP-1/CCR2 signaling. Consistently, ethanol increased mRNA levels of MCP-1 but not CCR2 (Fig. 2b). Since microglia are the major source of MCP-1, we examined the effect of ethanol on a cultured microglial cell line (SIM-A9). Ethanol drastically increased the expression of MCP-1 but not CCR2 in SIM-A9 cells (Fig. 2c).

### Bindarit and RS504393 block ethanol-induced caspase-3 activation in the developing brain

**Fig. 3:**
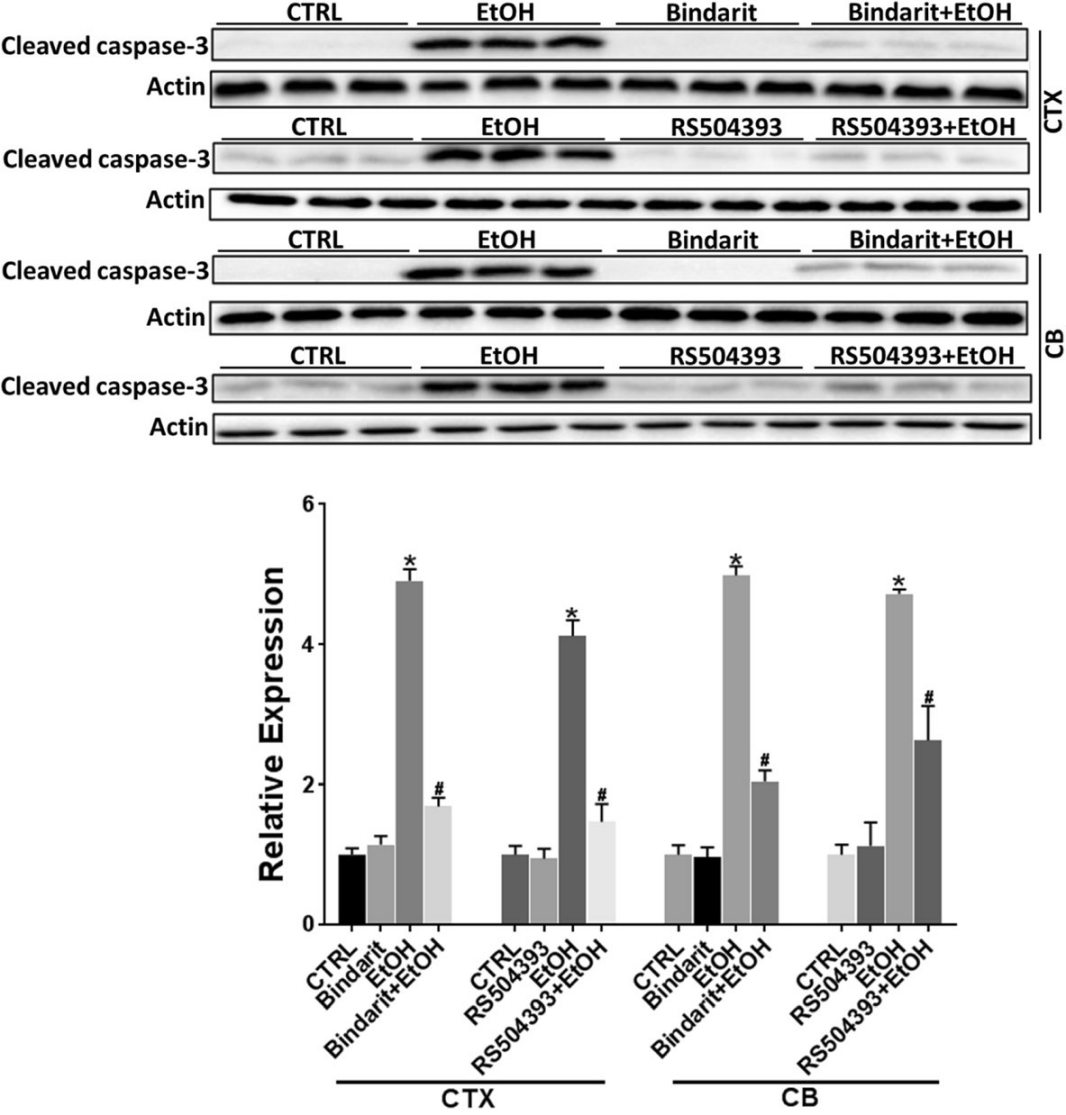
Effect of Bindarit and RS504393 on ethanol-induced apoptotic neuronal death in the developing mouse brain. Bindarit (100 mg/kg) or RS504393 (1 mg/kg) was administered by two subcutaneous (SC) injections at 24 and 0.5 h prior to EtOH exposure as described in the “Methods” section. 8 h after EtOH injection, the pups were sacrificed and the brains were harvested. The expression of cleaved caspase-3 in the cerebral cortex (CTX) and cerebellum (CB) was determined by IB and quantified by the normalization to actin. Each data point was the mean ± SEM of six animals. *p < 0.05, statistically significant difference from control group; # *p* < 0.05, statistically significant difference from EtOH-treated group

In this model of postnatal ethanol exposure, ethanol caused a wide-spread neuroapoptosis in the developing brain, which was indicated by the expression of cleaved caspase-3 [2, 24, 25]. As shown in Fig. 3, a significant alteration in cleaved caspase-3 was observed in the cortex [F(5,30) = 59.56; *p* ≤ 0.05] and cerebellum [F(5,30) = 48.18; *p* ≤ 0.05]. Ethanol increased the expression of cleaved caspase-3 in the cerebral cortex and cerebellum. Bindarit or RS504393 treatment significantly decreased ethanol-induced caspase-3 activation. There was little caspase-3 activation in the saline-injected controls and in Bindarit- or RS504393-treated groups.

### MCP-1^−/−^ and CCR2^−/−^ mice are more resistant to ethanol neurotoxicity

**Fig. 4:**
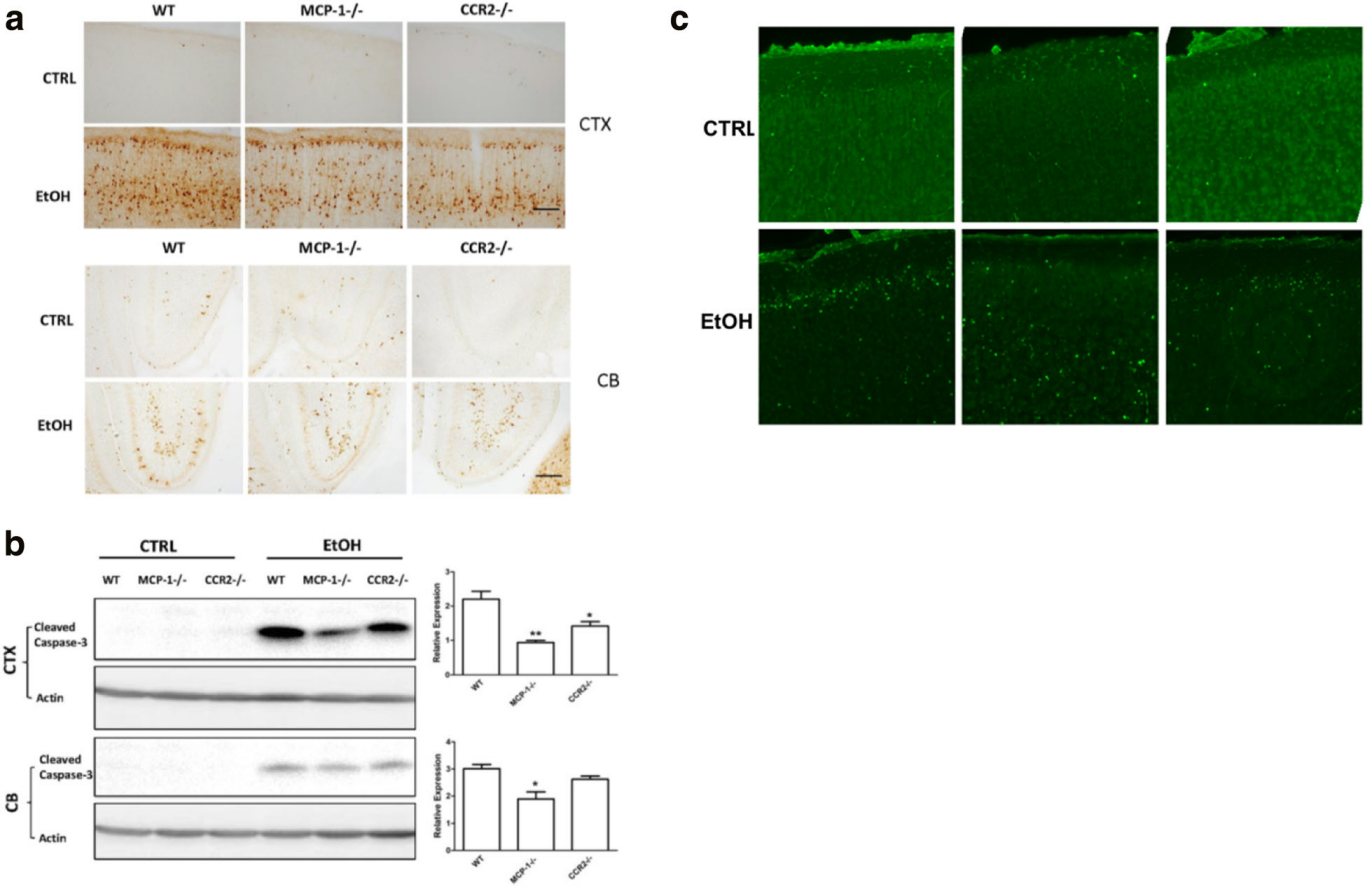
Effect of ethanol on the developing brain of wild-type, MCP-1^−/−^, and CCR2^−/−^ mice. Wild-type (WT), MCP-1^−/−^, and CCR2^−/−^ mice of PD4 were exposed to EtOH for 8 h. **a** The expression of cleaved caspase-3 in the cerebral cortex (CTX) and the cerebellum (CB) was examined by immunohistochemistry (IHC) as described in the “Methods” section. Bar = 100 μm. **b** The expression of cleaved caspase-3 in the CTX and CB was determined by IB and quantified by the normalization to the expression of actin. Each data point was mean ± SEM of 3–5 animals **p < 0.01, *p < 0.05, statistically significant difference from WT group. **c** The degenerating neurons in the visual cortex were demonstrated by Fluoro-Jade C staining as described in the “Methods” section. Bar = 100 μm

To verify the study using MCP-1 and CCR2 inhibitors, we compared ethanol-induced neuroapoptosis in the cortex and cerebellum among wild-type (WT), MCP-1^−/−^ mice, and CCR2^−/−^ mice. As shown in Fig. 4, ethanol increased the expression of cleaved caspase-3 in the cortex and cerebellum of WT mice; ethanol also increased the expression of cleaved caspase-3 in MCP-1^−/−^ mice and CCR2^−/−^ mice, but to a much lesser extent (Fig. 4a). It appeared that deletion of MCP-1 was more effective than CCR2 in terms of protective effects (Fig. 4b). The results from IB analysis confirmed that MCP-1^−/−^ and CCR2^−/−^ mice were less affected by ethanol-induced caspase 3 activation (Fig. 4b); F(2,33) = 16.57, *p* ≤ 0.05 for the cortex, and F(2,33) = 9.04, *p* ≤ 0.05, for the cerebellum. It appeared that CCR2 deletion did not offer protection in the cerebellum. MCP-1 and CCR2 deficiency-mediated protection against ethanol-induced neurodegeneration was confirmed by a decrease in Fluoro-Jade C-positive cells in MCP-1^−/−^ and CCR2^−/−^ mice, compared to WT mice following ethanol exposure (Fig. 4c).

### Bindarit and RS504393 alleviate ethanol-induced proinflammatory cytokines and microglial activation

**Fig. 5:**
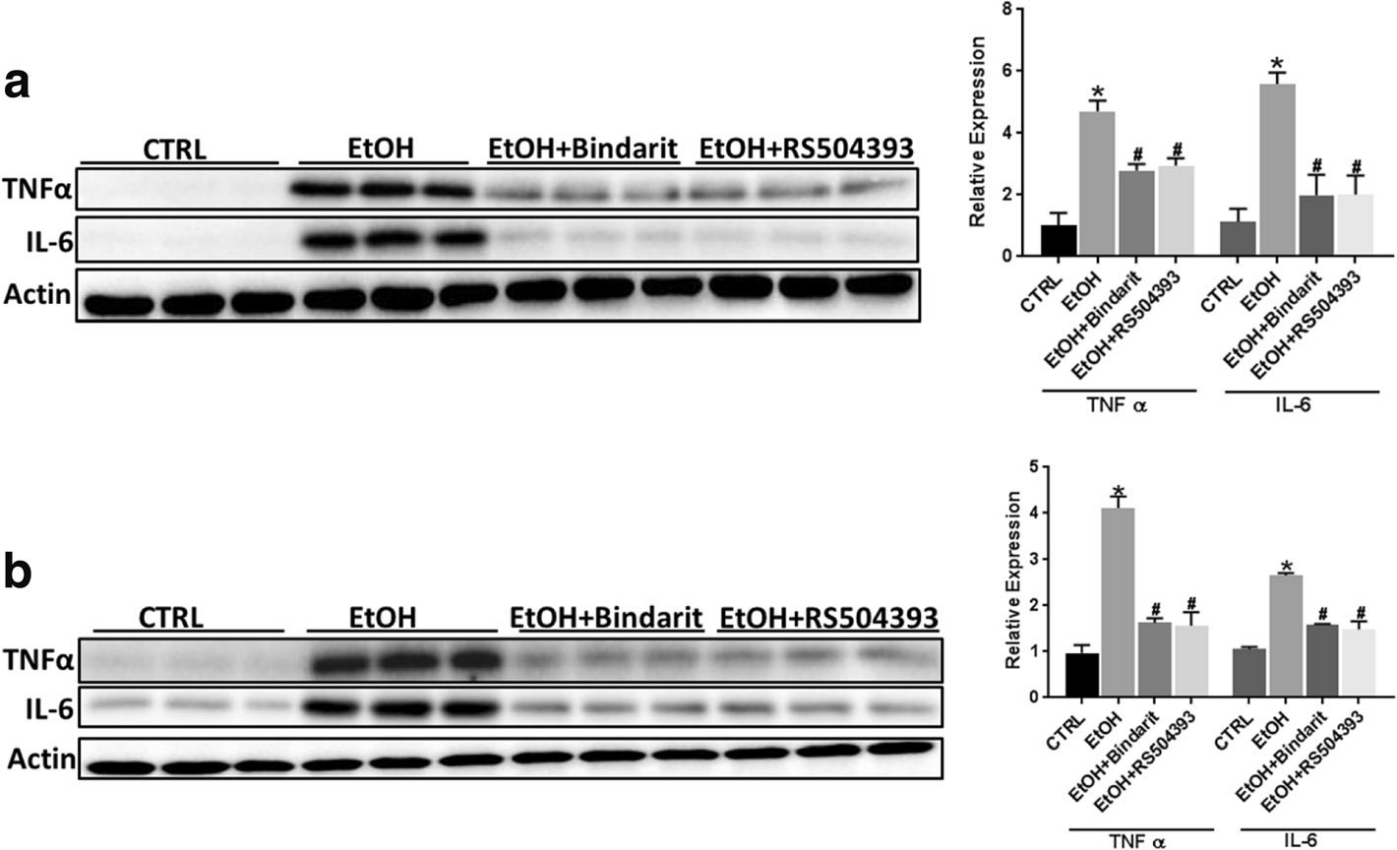
Effect of Bindarit and RS504393 on ethanol-induced inflammation in the developing brain and SIM-A9 cells. **a** Bindarit (100 mg/kg) or RS504393 (1 mg/kg) was administered by two subcutaneous (SC) injections at 24 and 0.5 h prior to EtOH exposure as described in the “Methods” section. 8 h after EtOH injection, the pups were sacrificed and the brains were harvested. The relative expression for proinflammatory cytokines IL-6 and TNFα in the brain of PD4 mice were determined with IB and quantified as described above. Each data point was the mean ± SEM of six animals. *p ≤ 0.05, statistically significant difference from control group; ^#^*p* ≤ 0.05, statistically significant difference from EtOH-treated group. **b** SIM-A9 cells were pretreated with Bindarit (300 μM) or RS504393 (100 μM) for 12 h prior to EtOH (0.4%) exposure. After ethanol exposure for 12 h, cells were harvested and processed for the expression of proinflammatory IL-6 and TNFα by IB as described above. Data are the mean ± SEM of three independent experiments. *p < 0.05, statistically significant difference from control group; ^#^*p* < 0.05, statistically significant difference from EtOH-treated group

**Fig. 6:**
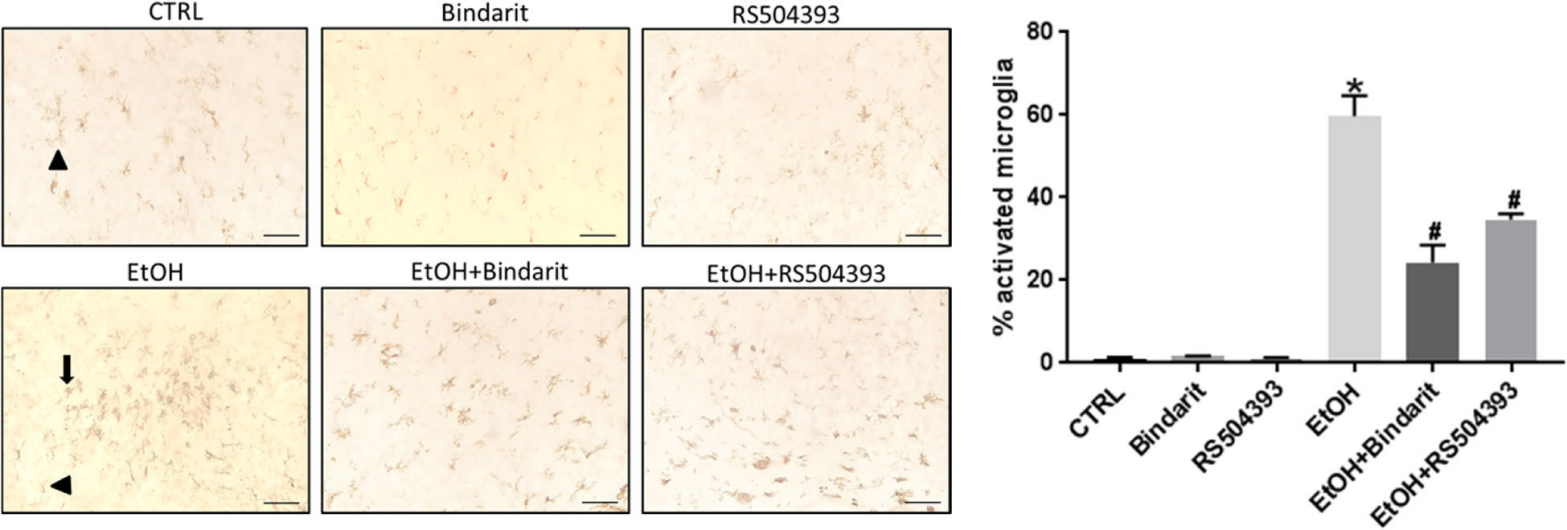
Effect of Bindarit and RS504393 on ethanol-induced microglial activation in the developing mouse brain. Bindarit (100 mg/kg) or RS504393 (1 mg/kg) was administered by two subcutaneous (SC) injections at 24 and 0.5 h prior to EtOH exposure as described above. 8 h after EtOH injection, the pups were sacrificed and the brains were harvested. A representative image of inferior colliculus is shown. Microglia were identified by IHC of Iba-1. The resting microglia exhibit long branching processes and a small cellular body, which are indicated by arrow heads; while the activated microglia have fewer but thicker processes with a larger cell body, which are indicated by arrows. The percentage of activated Iba-1 positive cells in total Iba-1 positive cells was calculated as described in the “Methods” section. Bar = 50 μm. Each data point was the mean ± SEM of three animals. *p < 0.05, statistically significant difference from control group, ^#^*p* < 0.05, statistically significant difference from EtOH-treated group

Ethanol-induced neuronal death is accompanied by neuroinflammation which may play a role in CNS damage. We sought to determine whether Bindarit or RS504393 can reduce ethanol-induced neuroinflammation. Interleukin-6 (IL-6) and tumor necrosis factor alpha (TNFα) are major pro-inflammatory cytokines that are involved in injuries in the CNS [46, 47]. Ethanol increased the expression of IL-6 and TNFα in the brain of PD4 mice and cultured SIM-A9 cells; Bindarit and RS504393 inhibited ethanol-induced increase of IL-6 [F(3,20) = 40.12; *p* ≤ 0.05], and TNFα [F(3,20) = 31.21; *p* ≤ 0.05] in the developing brain (Fig. 5a) and in cultured SIM-A9 cells [IL6, F(3,8) = 29.31; p ≤ 0.05; TNFα, F(3,8) = 30.54; *p* ≤ 0.05] (Fig. 5b). Pathological activation of microglia is involved in neuroinflammation and neurodegeneration in various CNS disorders [48–51]. We therefore examined the effect of Bindarit or RS504393 on ethanol-induced microglia activation. Microglial activation was assessed morphologically by IHC of Iba-1. Ethanol significantly increased the number of active microglia; Bindarit and RS504393 inhibited ethanol-induced activation of microglia [F(5,12) = 200.63; *p* ≤ 0.05] (Fig. 6).

### Bindarit and RS504393 protect neurons against ethanol-induced neuronal death in co-cultures of primary neurons and microglia cells

**Fig. 7:**
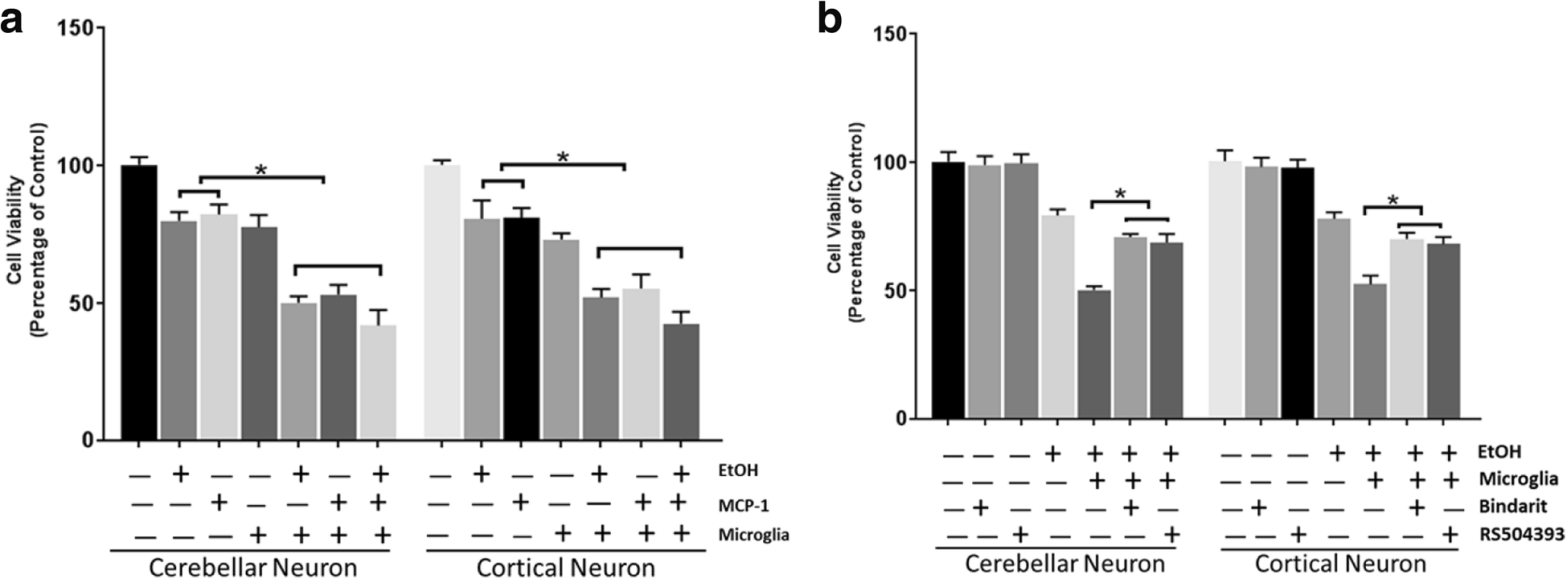
Effect of Bindarit and RS504393 on ethanol-induced neuronal death in the co-cultures of microglia/primary neurons. **a** Co-cultures of SIM-A9 cells and primary cortical neurons/cerebellar neurons were established described in the “Methods” section. The co-cultures were treated with MCP-1 (10 ng/ml in PBS) or EtOH (0.4%) for 48 h. The viability of neurons was determined by MTT as described in the “Methods” section. Data were the mean ± SEM of three independent experiments. *p < 0.05, statistically significant difference from primary neuronal culture alone. **b** SIM-A9 cells were treated with Bindarit (300 μM) or RS504393 (100 μM) for 12 h. After that, the inserts containing SIM-A9 cells were placed in the culture wells containing cortical neurons or cerebellar neurons. The co-cultures were then treated with EtOH (0.4%) for 48 h, and the viability of neurons was determined by MTT. Each data point was the mean ± SEM of three independent experiments. *p < 0.05, statistically significant difference from co-cultures without Bindarit or RS504393 treatment

Since inhibition of MCP-1/CCR2 signaling alleviated ethanol-induced neuroapoptosis and microglial activation in the developing brain, we hypothesized that MCP-1/CCR2-regulated microglial activation may contribute to neuronal death. To test this hypothesis, we used a co-culture system in which primary neurons (mouse cortical neurons or cerebellar neurons) and microglial cells (SIM-A9 cells) were cultured together, allowing communication without direct contact. As shown in Fig. 7a, ethanol- and MCP-1-induced neuronal death was greater in microglia/neuron co-cultures than in cerebellar neuron cultures [F(6,14) = 34.18, *p* ≤ 0.05] or cortical neuron cultures [F(6,14) = 32.45, *p* ≤ 0.05] alone. Bindarit and RS504393 significantly ameliorated ethanol-induced neuronal death in the microglia/cerebellar neuron co-cultures [F(6,14) = 28.71, *p* ≤ 0.05], and microglia/cortical neuron co-cultures [F(6,14) = 26.19, p ≤ 0.05] (Fig. 7b).

### TLR4 and GSK3β mediate ethanol-induced proinflammatory cytokines in microglial cells

**Fig. 8:**
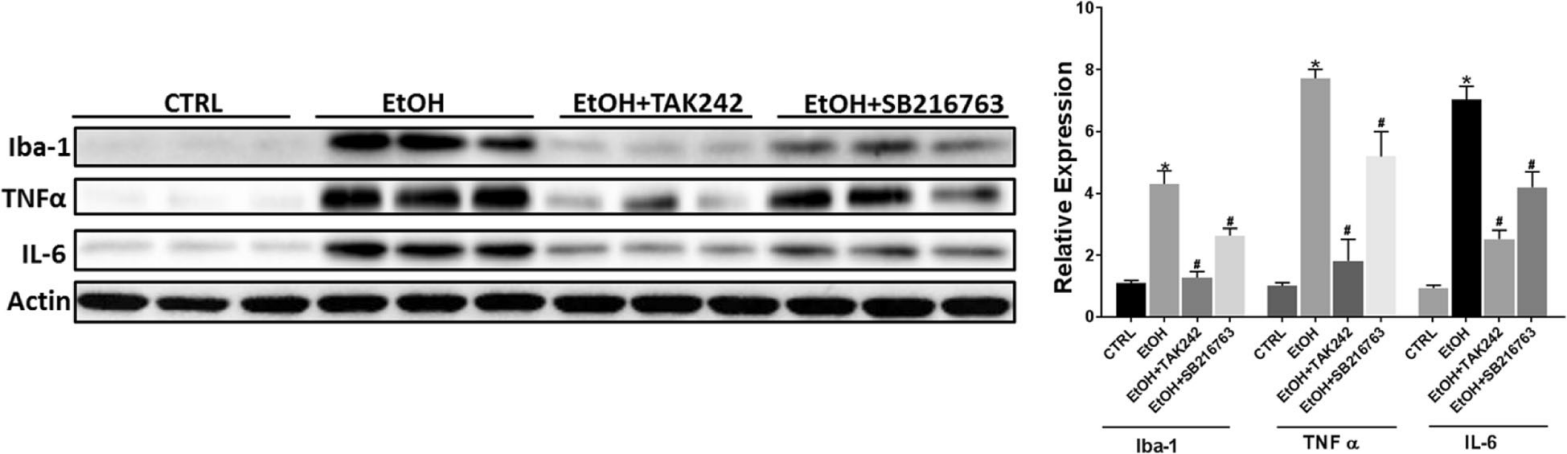
Role of TLR4 and GSK3β in EtOH-induced inflammation. SIM-A9 cells were pretreated with TAK242 (1 μM) or SB-216763 (10 μM) for 12 h, then exposed to EtOH (0.4%) for 12 h. The expression of Iba-1, TNFα, and IL-6 was determined by IB and quantified by the normalization to the expression of actin. Each data point the mean ± SEM of three independent experiments. *p < 0.05, statistically significant difference from control group; #*p* < 0.05, statistically significant difference from EtOH-treated group

**Fig. 9:**
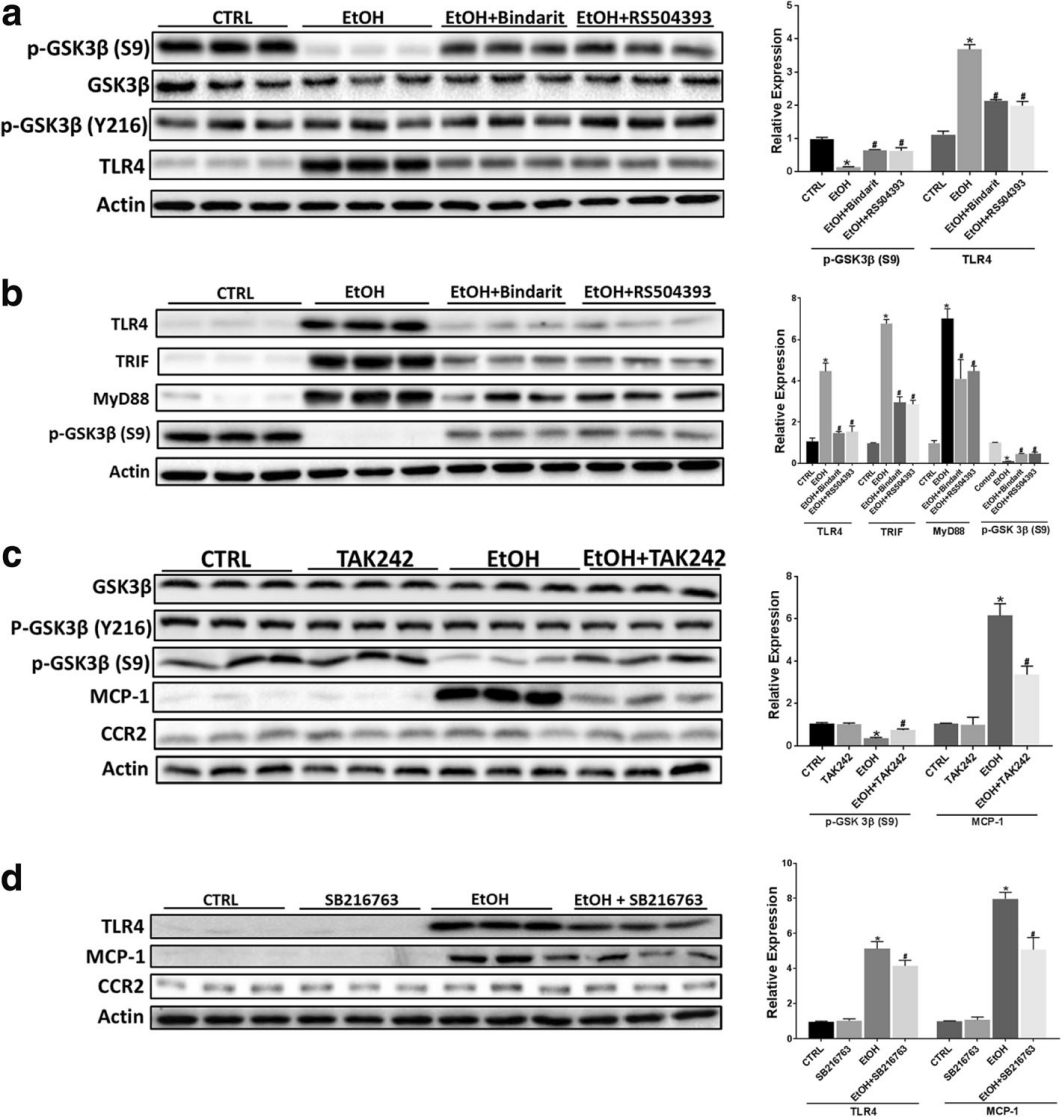
The interaction of MCP-1/CCR2 signaling, TLR4 and GSK3β in response to ethanol exposure. **a** SIM-A9 cells were pretreated with RS504393 (100 μM) or Bindarit (300 μM) for 12 h, and then exposed to EtOH (0.4%) for 12 h. The expression of GSK3β, phosphorylated GSK3β at serine 9 (phospho-GSK3β^S9^), phosphorylated GSK3β at tyrosine 216 (phospho-GSK3β^Y216^), and TLR4 were determined by IB and quantified by the normalization to the expression of actin. **b** C57BL6 mice of PD4 were treated with Bindarit, RS504393, and EtOH as described in Fig. 2. 8 h after EtOH treatment, the expression of TLR4 and its downstream adaptors TRIF and MyD88, and phospho-GSK3β^S9^ in the brain was determined by IB and quantified by the normalization to the expression of actin. **c** SIM-A9 cells were pretreated with TAK242 (1 μM) for 12 h, and then exposed to EtOH (0.4%) for 12 h. The expression of GSK3β, phospho-GSK3β^S9^, phospho-GSK3β^Y216^, MCP-1, and CCR2 was by IB and quantified by the normalization to the expression of actin. **d** SIM-A9 cells were pretreated SB-216763 (10 μM) for 12 h, and then exposed to EtOH (0.4%) for 12 h. The expression of TLR4, MCP-1, and CCR2 was by IB and quantified by the normalization to the expression of actin. Each data point was the mean ± SEM of three independent experiments. *p < 0.05, statistically significant difference from control group; #*p* < 0.05, statistically significant difference from EtOH-treated group

It is well established that TLR4 and its downstream adaptors TRIF and MyD88 regulate microglial activation and production of inflammatory mediators [52–54]. GSK3β is also a key mediator of microglial activation and neuroinflammation [55–57]. MCP-1 was shown to activate GSK3β signaling in human breast carcinoma cells [58]. We hypothesized that the interaction among MCP-1/CCR2, TLR4, and GSK3β may mediate ethanol-induced microglial activation and expression of proinflammatory factors. We first determined whether TLR4 and GSK3β were involved in ethanol-induced expression of proinflammatory cytokines. We pre-treated SIM-A9 cells with either TLR4 inhibitor TAK242 or GSK3β inhibitor SB216763 to block TLR4 and GSK3β signaling prior to ethanol exposure. As shown in Fig. 8, TAK242 and SB216763 drastically attenuated ethanol-induced increase in the expression of Iba-1 [F(3,8) = 40.13, *p* ≤ 0.05], IL-6 [F(3,8) = 56.78, *p* ≤ 0.05], and TNF-α [F(3,8) = 65.17, *p* ≤ 0.05]. We next sought to determine whether MCP-1/CCR2 signaling was involved in ethanol-activated GSK3β and TLR4. The activity of GSK3β is mainly regulated by the phosphorylation at serine 9 which results in the inhibition of GSK3β [59–61], conversely, the phosphorylation at tyrosine 216 positively regulated its activity [62]. As shown in Fig. 9a, ethanol activated GSK3β by reducing the phosphorylation at Ser9 in SIM-A9 cells; blocking MCP-1/CCR2 signaling by Bindarit or RS504393 attenuated ethanol-induced dephosphorylation of GSK3β (Ser9) [F(3,8) = 50.87, *p* ≤ 0.05]. Blocking MCP-1/CCR2 signaling also inhibited ethanol-induced TLR4 (F(3,8) = 35.27; *p* < 0.05). Animal studies confirmed these findings and showed that Bindarit or RS504393 treatment attenuated ethanol-induced dephosphorylation of GSK3β (Ser9) [F(3,8) = 59.54; *p* ≤ 0.05] and upregulation of TLR4 [F(3,8) = 52.18; *p* ≤ 0.05], MyD88 [F(3,8) = 57.15, *p* ≤ 0.05], and TRIF [F(3,8) = 63.17; *p* ≤ 0.05] in the brain of PD4 mice (Fig. 9b).

We then investigated the interaction between TLR4 and GSK3β in response to ethanol exposure. Inhibition of TLR4 signaling by TAK242 attenuated ethanol-induced dephosphorylation of GSK3β (Ser9) [F(3,8) = 19.98; *p* ≤ 0.05] and upregulation of MCP-1 [F(3,8) = 56.78; *p* ≤ 0.05] in SIM-A9 cells (Fig. 9c). On the other hand, blocking GSK3β activation by SB216763 inhibited ethanol-induced increase of MCP-1 [F(3,8) = 70.35; *p* ≤ 0.05] and TLR4 [F(3,8) = 42.36; *p* ≤ 0.05](Fig. 9d). The results indicated that there was considerable interaction among MCP-1/CCR2 signaling, TLR4, and GSK3β in response to ethanol exposure.

## Discussion

In this study, we showed that ethanol increased the expression of MCP-1 but not CCR2 in the brain of PD4 mice and microglia cells (SIM-A9). MCP-1 synthesis inhibitor Bindarit and CCR2 antagonist RS504393 inhibited ethanol-induced neuroapoptosis, microglial activation, and the expression of pro-inflammatory factors. Further studies using gene knock out mice confirmed that the deficiency in MCP-1 or CCR2 made mice more resistant to ethanol-induced neurodegeneration. Moreover, ethanol and MCP-1 caused more neuronal death in neuron/microglia co-cultures than neuronal culture alone. Blocking MCP-1/CCR2 signaling protected primary cortical and cerebellar neurons against ethanol-induced death in the neuron/microglia co-cultures. It appeared that TLR4 and GSK3β mediated ethanol-induced microglial activation and pro-inflammatory cytokines in cultured microglia cells, and there was considerable interaction among TLR4, GSK3β, and MCP-1/CCR2 signaling in response to ethanol exposure.

The mechanisms underlying ethanol-induced neurodegeneration in the developing brain are complex. Multiple mechanisms may be involved; these include oxidative stress [63, 64], endoplasmic reticulum (ER) stress [25], and interference of signaling by neurotrophic factors and disruption of microRNAs [65]. Recent evidence indicates that neuroinflammation plays an important role in the pathogenesis of FASD and AUD [12, 66, 67]. Ethanol-induced neurodegeneration in both adult and developing brain is accompanied by microglial activation and neuroinflammation [12, 66, 68, 69]. We have recently shown that inhibition of microglial activation and neuroinflammation by minocycline offered protection against ethanol-induced neurodegeneration in the developing brain [41], suggesting that microglial activation and neuroinflammation may contribute to ethanol neurotoxicity in the immature CNS.

MCP-1 is a key chemokine involved in neuroinflammation. In mouse and human brain, MCP-1 and its receptor CCR2 are primarily expressed by microglia [8]. MCP-1/CCR2 signaling is involved in numerous neuroinflammatory diseases, such as multiple sclerosis, stroke, and Alzheimer’s disease [14–19]. Chronic ethanol exposure induces MCP-1 expression in adult human and mice brain [24, 25]. We have recently shown that ethanol stimulated MCP-1/CCR-2 signaling in the spinal cord of early postnatal mice, and knocking out MCP-1 or CCR2 made mice resistant to ethanol-induced apoptosis of spinal cord neurons [70], indicating the involvement of MCP-1/CCR2 signaling in ethanol-induced neurodegeneration in the developing spinal cord.

The current study showed that ethanol upregulated MCP-1 at both mRNA and protein levels but not CCR2 expression in the developing brain. Ethanol stimulation of MCP-1/CCR2 signaling was confirmed by an upregulation of the downstream effector, MCPIP (Fig. 2). Disrupting MCP-1/CCR2 signaling by either MCP-1 synthesis inhibitor or CCR2 antagonist made mice less susceptible to ethanol-induced apoptotic cell death, microglial activation, and the expression of pro-inflammatory factors (Figs. 3, 4, 5, and 6). Gene deletion of MCP-1 or CCR2 also offered protection against ethanol-induced neurodegeneration in the developing brain (Fig. 4). It appears that MCP-1 deletion provided better protection than CCR2 deletion (Fig. 4b). The underlying mechanisms are currently unknown. It is possible that MCP-1 may interact with other chemokine receptors than CCR2 in microglia. It is reported that MCP-1 binds to CCR5 in osteoblastic cells [71]. MCP-1/CCR2 signaling plays an important role in microglial activation and neuroinflammation [8–10, 72]. Therefore, the neuroprotection caused by the disruption of MCP-1/CCR2 signaling may result from suppression of microglial activation and neuroinflammation. Using a neuron/microglia co-culture system, we demonstrated that the presence of microglial cells indeed exacerbated ethanol-induced neuronal death; while blocking MCP-1/CCR2 signaling attenuated enhanced ethanol neurotoxicity in this neuron/microglia co-culture system (Fig. 7). The result further supported the hypothesis that the activation of microglia contributed to ethanol neurotoxicity. Although our findings suggest that MCP-1/CCR2-mediated microglial activation and neuroinflammation is involved in ethanol neurotoxicity, we could not rule out the possibility of ethanol directly killing the neurons. In primary neuronal cultures alone, ethanol also reduced neuronal viability but to a lesser extent, compared to neuron/microglia co-cultures (Fig. 7). It is possible that in the brain, ethanol initially causes neuronal damage, triggering MCP-1/CCR2-mediated microglial activation and neuroinflammation, which further exacerbates neurodegeneration.

Although MCP-1 and CCR2 are primarily expressed by microglia in mouse and human brain, they are also expressed in other cell types in the brain, such as astrocytes and neurons, but to a lesser extent [8, 73, 74]. Therefore, the situation in vivo is complex and it is difficult to separate the effects of microglia-derived MCP-1 from that deprived from other cell types unless microglia-specific MCP-1 or CCR2 knock out mice are available.

TLR4, GSK3β, and p38 MAPK are important regulators of microglial activation and neuroinflammation [44, 52, 54, 75, 76]. Since ethanol had little effect on p38 MAPK in the developing brain [41], we focused on TLR4 and GSK3β in this study. Indeed, TLR4 and GSK3β were activated by ethanol and involved in ethanol-induced microglial activation and upregulation of proinflammatory cytokines (Figs. 8 and 9). Ethanol activated GSK3β by inducing dephosphorylation of GSK3β at Ser9 in vitro and in vivo (Fig. 9a, b). This is consistent with our previous findings that ethanol decreased the phosphorylation of GSK3β (Ser9) in the developing brain and cultured neuronal cells [60, 77]. Blocking MCP-1/CCR2 signaling partially mitigated ethanol-induced dephosphorylation of GSK3β (Ser9), therefore alleviating ethanol-mediated activation of GSK3β. Similarly, blocking MCP-1/CCR2 signaling effectively inhibited ethanol-induced upregulation of TLR4 in vitro and in vivo (Fig. 9a, b). These results suggest that MCP-1/CCR2 signaling was involved in ethanol-induced activation of GSK3β and TLR4.

**Fig. 10:**
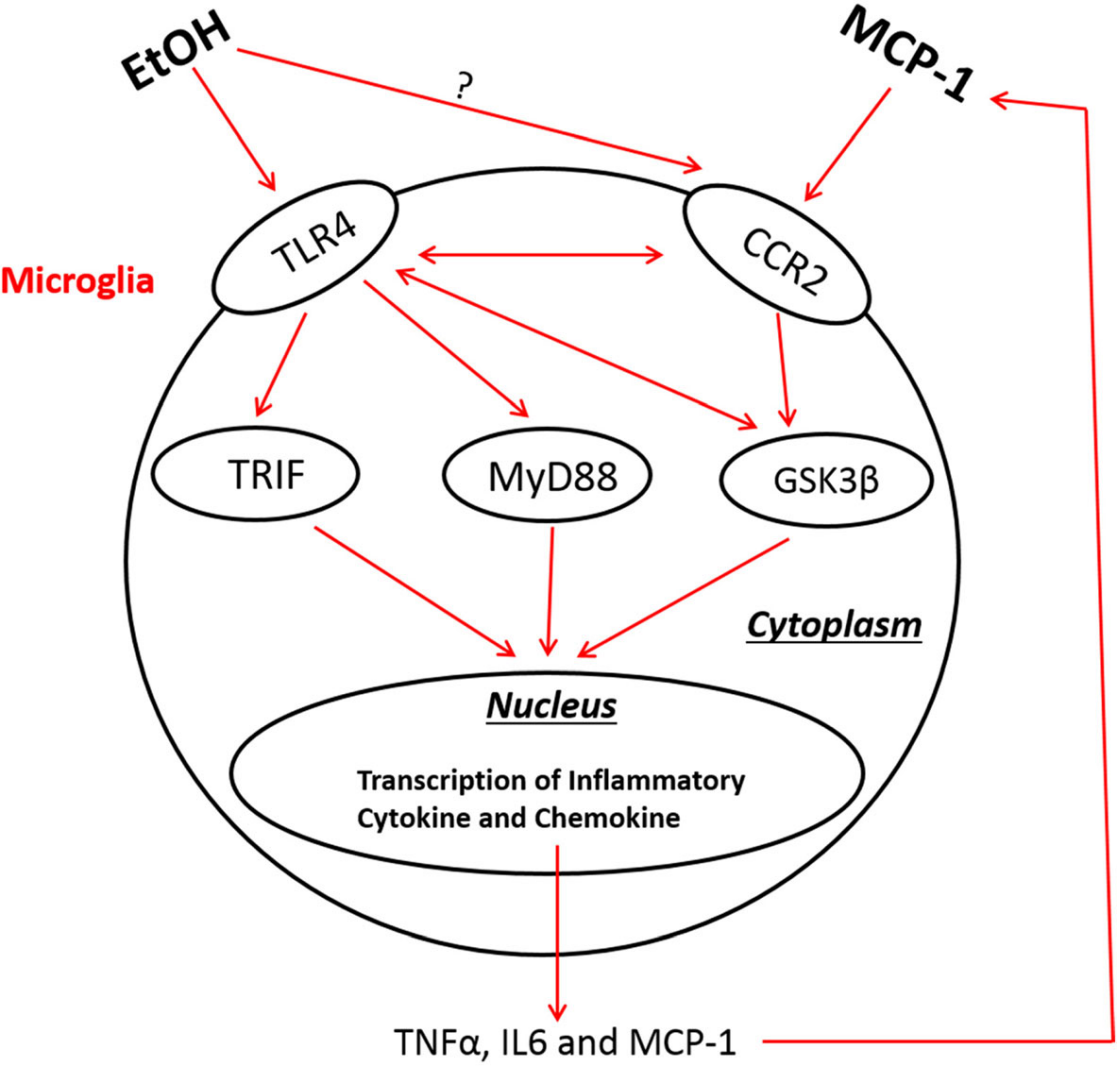
The interaction among MCP-1/CCR2 signaling, TLR4, and GSK3β in ethanol-induced neuroinflammation. EtOH may stimulate TLR4 and possibly CCR2, and activate the downstream effectors, such as TRIF and MyD88. Active TLR4 may also activate GSK3β which may further stimulate TLR4 pathway. As a result, the activation of TRIF, MyD88, and GSK3β stimulates transcription factors which upregulate the expression of proinflammatory cytokines and chemokines (e.g., TNF-α, IL6, and MCP-1). The released MCP-1 interacts with CCR2 and further activates TLR4 and GSK3β; the positive feedback loop intensifies the neuroinflammation that is toxic to neurons in the developing CNS

It appears that there was considerable interaction among MCP-1/CCR2 signaling, GSK3β, and TLR4 in response to ethanol exposure. Blocking TLR4 by TAK242 alleviated ethanol-induced dephosphorylation of GSK3β (Ser9) and partially inhibited ethanol-induced upregulation of MCP-1 (Fig. 9c). On the other hand, blocking GSK3β also attenuated ethanol-induced upregulation of MCP-1 and TLR4 (Fig. 9d). Based on these findings, we propose a model to illustrate the cascade of ethanol-induced microglial activation and neuroinflammation (Fig. 10). In this model, ethanol could directly or indirectly activate TLR4 and stimulate its downstream effectors such as TRIF and MyD88; the active TLR4 may also activate GSK3β which may further stimulate TLR4. As a result, the activation of TRIF, MyD88, and GSK3β stimulates transcription factors, such as AP-1 and NF-κB which upregulate the expression of proinflammatory cytokines and chemokines (e.g., TNF-α, IL6, and MCP-1). The released MCP-1 interacts with CCR2 and further activates GSK3β and TLR4; the positive feedback loop intensifies the neuroinflammation toxic to neurons. It is also possible that ethanol could directly activate CCR2 and initiate the downstream signaling cascades. This possibility is worthy of further investigation.

There are several important implications from this study. The current finding establishes an important role of GSK3β in microglial activation and neuroinflammation. We have previously demonstrated that ethanol-induced activation of GSK3β in neurons may cause neurodegeneration in the developing brain and in vitro [78]. Our recent study showed that minocycline inhibited GSK3β and offered protection against ethanol neurotoxicity in the developing brain [41]. Together, these findings suggest that the activation of GSK3β in both neurons and microglia contributes to ethanol neurotoxicity, although it may operate by different mechanisms. It further supports that both neuronal and microglial GSK3β are valid therapeutic targets for the treatment of ethanol neurotoxicity in the developing CNS.

In addition to the microglial activation and neuroinflammation, stimulation of MCP-1/CCR2 signaling may cause neurotoxicity through other mechanisms. For example, the activation of MCP-1/CCR2 signaling is reported to cause ER stress through the upregulation of MCPIP in cardiomyocytes and osteoclasts [79]. Kim et al. showed that CCR2 inhibitor attenuated ER stress and decreased the expression of inflammatory cytokines in the liver of type 2 diabetic mice [80]. ER stress has recently been proposed as an important mechanism for ethanol-induced damage to the CNS [25, 81]. Ethanol-induced ER stress in the developing spinal cord is significantly reduced in MCP-1^−/−^ and CCR2^−/−^mice [70]. Therefore, it is also likely that ethanol-induced upregulation of MCP-1 may cause ER stress which exacerbates neurodegeneration in the developing CNS.

MCP-1/CCR2 signaling has been implicated in AUD. For example, it is shown that MCP-1/CCR2 signaling regulates voluntary ethanol consumption. MCP-1 is also involved in ethanol-induced anxiety-like behavior in adolescent rats [82]. The current finding along with our recent study [70] add that MCP-1/CCR2-mediated neuroinflammation and microglial activation contribute to ethanol neurotoxicity in the developing CNS. Thus, MCP-1/CCR2 signaling is likely also involved neurological deficits in associated with FASD.

## Conclusion

In conclusion, MCP-1/CCR2 signaling played an important role in ethanol-induced microglial activation/neuroinflammation and neurodegeneration in the developing brain. The findings offer a potential new therapeutic avenue for FASD by targeting MCP-1/CCR2 signaling pathway.

## Abbreviation

AD: Alzheimer’s disease
AUD: Alcohol use disorders
CCR2: Chemokine (C–C motif) receptor 2
CNS: Central nervous system
ER: Endoplasmic reticulum
FASD: Fetal alcohol spectrum disorder
GSK3β: Glycogen synthase kinase 3 beta
Iba-1: Ionized calcium binding adaptor molecule 1
IFN-γ: Interferon-γ
IHC: Immunohistochemistry
IL: Interleukin
MCP-1: Monocyte chemoattractant protein-1
PD: Parkinson’s disease
TLR4: Toll-like receptor 4
TNF-α: Tumor necrosis factor-α
